# Preparation and Characterization of Camellia Oil Microcapsules Using Spray Drying Coupled with Sodium Caseinate/Xanthan Gum-Stabilized Emulsion Template

**DOI:** 10.3390/foods14213610

**Published:** 2025-10-23

**Authors:** Lihua Zhang, Lala Li, Yingying Xin, Jiawei Xue, Zhenwei Li, Bakht Ramin Shah, Wei Xu

**Affiliations:** 1 College of Life Science, Xinyang Normal University, Xinyang 464000, China; lihuazhang25@163.com (L.Z.); 15517984382@163.com (L.L.); 18847015570@163.com (Y.X.); 16639008867@163.com (J.X.); 13673465788@163.com (Z.L.); 2 DRIFT-FOOD Centre, Faculty of Agrobiology, Food and Natural Resources, Czech University of Life Sciences Prague, 16500 Prague, Czech Republic; raminshah83@gmail.com

**Keywords:** camellia oil, microcapsule, spray drying, xanthan gum, β-carotene

## Abstract

To enhance the high-value utilization of camellia oil and innovation in functional foods, this study developed a stable emulsion template using xanthan gum (XG) and sodium caseinate (CAS) for the preparation of camellia oil microcapsules via spray drying. Employing scanning electron microscopy (SEM), Fourier transform infrared spectroscopy (FTIR), and thermogravimetric analysis (TGA), alongside additional analytical methods, this study systematically examined the influence of drying temperature (145 °C, 165 °C, and 185 °C) and XG concentration (0.2%, 0.3%, and 0.4%) on the physicochemical properties and functional attributes of the microcapsules. Results indicated that 0.3% XG was the optimal concentration, enabling uniform emulsion droplet dispersion while balancing microcapsule bulk density and solubility, thereby optimizing processing and dissolution properties. 165 °C was identified as the optimal drying temperature, yielding the highest microcapsule yield (53.68%), moisture content (<2.84%) meeting storage standards, and optimal β-carotene encapsulation efficiency (89.6%) and DPPH radical scavenging rate (74.80 ± 0.34%). FTIR analysis confirmed successful encapsulation of camellia oil within microcapsules. TGA and in vitro digestion experiments demonstrated excellent thermal stability and digestive characteristics of the microcapsules. In summary, this study identified the most favorable preparation conditions for camellia oil microcapsules, providing theoretical support and technical reference for expanding camellia oil applications in the food industry.

## 1. Introduction

Camellia oil, also known as camellia seed oil, is a high-quality edible oil extracted from the seeds of the camellia plant [1]. Its fatty acid profile and physicochemical properties closely resemble those of olive oil, which has led to its recognition as the ‘Eastern olive oil’ and one of the highest-quality edible oils [2]. Research has confirmed that camellia oil contains a high concentration of bioactive constituents, including polyphenols, unsaturated fatty acids, squalene, vitamin E, and phytosterols [3]. These constituents demonstrate a range of pharmacological activities, encompassing cholesterol reduction and prevention of cardiovascular diseases, as well as anti-inflammatory, antibacterial, antitumor, and antioxidant properties [4]. However, these constituents are highly sensitive to heat, oxygen, and light during processing and storage, potentially leading to changes in nutritional content and food quality [5]. To overcome the inherent instability and poor dispersibility of vegetable oils in food systems, emulsion-based strategies are widely adopted. Emulsions achieve strong resistance to droplet coalescence through the adsorption of particles at the oil–water interface, where electrostatic and steric repulsion forces help maintain interfacial stability [6]. Moreover, they pose minimal toxicity to humans and cause no environmental pollution, making them increasingly attractive [7].

Microencapsulation serves to shield active ingredients from harsh environmental conditions by enclosing them within a polymeric shell at the micron scale. In the food industry, spray drying represents the most commonly employed technique for producing microcapsules [8]. This method not only shortens processing time compared to freeze-drying but also promotes the integration of active components into the wall matrix, minimizing their presence on the particle surface. Spray drying effectively addresses the quality deterioration of unsaturated oils, as the wall material efficiently prevents core material exposure to oxidants, significantly enhancing the storage stability of oxidizable cores [9]. Recent studies further suggest that using an emulsion template during spray drying helps lower surface oil content and raises encapsulation efficiency. Compared to free-form oils, microencapsulated oils exhibit enhanced oxidative stability, improved bioavailability, and even controllable release efficacy [10]. As a result, microencapsulation offers a practical route to enhance oil handling and stability, thereby broadening its use in food applications.

Sodium caseinate (CAS), a water-soluble derivative of casein, shows stronger emulsifying capacity than many plant-derived proteins such as those from soy or pea [11]. Xanthan gum (XG) is a microbial polysaccharide produced by aerobic fermentation of *Xanthomonas campestris* [12], characterized by its high viscosity, shear-thinning behavior, and stability under high temperatures and varying pH conditions. In the food industry, it is frequently employed as a thickening and stabilizing agent and has been shown to synergistically improve emulsion stability when used in conjunction with proteins [13].

Therefore, this study employs XG- and CAS-stabilized emulsion templates to prepare camellia oil microcapsules via spray drying. The physicochemical properties of these microcapsules are analyzed, and their potential for nutrient delivery is thoroughly investigated. This research aims to provide new directions for the high-value utilization of camellia oil and the innovation of functional foods, while simultaneously advancing the deepening of camellia oil microcapsule-related studies and accelerating their industrial transformation.

## 2. Materials and Methods

### 2.1. Materials

Sodium caseinate (CAS, protein content ≥ 90%) was purchased from Sinopharm Chemical Reagent Co., Ltd. (Shanghai, China). Xanthan gum (XG) was purchased from Shanghai Yuanye Biotechnology Co., Ltd. (Shanghai, China). Maltodextrin (MD) was purchased from Anhui Cool Bioengineering Co., Ltd. (Huaibei, China). β-Carotene was purchased from TCI (Shanghai) Chemical Industry Development Co., Ltd. (Shanghai, China). Food-grade camellia oil was purchased from Henan Luda Camellia Oil Co., Ltd. (Xinyang, China). Ultra-pure water was obtained from a Milli-Q water purification system (Millipore, Burlington, MA, USA), and all other chemicals were of analytical grade.

### 2.2. Preparation of Camellia Oil Emulsion

Specified amounts of CAS and XG were separately dissolved in ultrapure water. After stirring for 4–6 h, a 1% CAS solution and XG solutions at different concentrations (0.2%, 0.3%, and 0.4%, *w*/*w*) were obtained. Equal volumes of the two solutions were then mixed and stirred for 3 h. MD was added to achieve a total solids content of 10%. Finally, 10% (by mass) camellia oil was incorporated into the composite solution, and the mixture was homogenized using an IKA Ultra-Turax T18 homogenizer (IKA Works (Guangzhou) Co., Ltd., Guangzhou, China) at 15,000 rpm for 3 min to form the final emulsion.

### 2.3. Rheological Analysis of Camellia Oil Emulsion

The steady-state rheological properties of the camellia oil emulsion were measured on a rotational rheometer (DHR-2, TA, New Castle, DE, USA). The tests employed 40 mm aluminum parallel plates with a 1 mm gap, conducting shear flow experiments at 20 °C by increasing the shear rate from 0.1 to 100 s^−1^. The resulting shear flow curves were fitted to the power law model:

$$\eta \;=\;k{\;\times \;\gamma }^{n-1}$$

In the equation, k represents the consistency index (Pa·s), *γ* represents the shear rate (s^−1^), and n represents the flow index.

### 2.4. Confocal Laser Scanning Microscopy of Camellia Oil Emulsion

The microstructural characteristics of the emulsion were examined utilizing a laser confocal scanning microscope (SP8, Leica, Wetzlar, Germany). One milliliter of undried sample was mixed with 40 μL of mixed dye solution (0.1% Nile red and 0.1% Nile blue in isopropanol) and incubated at room temperature for 10 min. Subsequently, the sample was examined for microstructure using a 40× objective lens. Excitation wavelengths of 488 nm and 633 nm were used for Nile red and Nile blue, respectively.

### 2.5. Preparation of Camellia Oil Microcapsules

**Figure 1:**
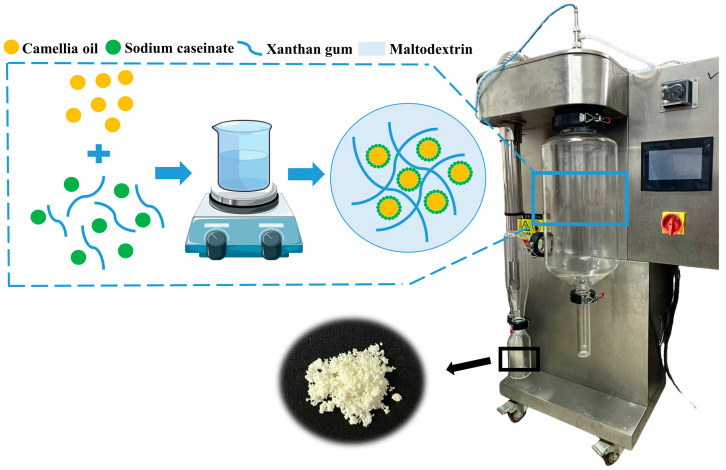
Schematic diagram of camellia oil emulsion and microcapsule preparation.

The prepared camellia oil emulsion was moderately diluted with ultrapure water to achieve better atomization. The diluted emulsion was then delivered via a peristaltic pump to a spray dryer (BILON-6000Y, Shanghai, China) for spray drying (inlet temperatures of 145 °C, 165 °C, and 185 °C, peristaltic pump speed of 5 mL/min, and air pressure of 0.1 MPa) to obtain camellia oil microcapsules (Figure 1).

### 2.6. Color Variation in Camellia Oil Microcapsules

The color parameters L* (lightness), a* (redness), and b* (yellowness) were recorded for the microcapsules employing a colorimeter (Hangzhou Caipu, CS-580, Hangzhou, China), with three repeated measurements per sample. Take the average of the readings and calculate the total color difference (ΔE) using the following formula:

$$\Delta E\;=\;\sqrt{{\left(\Delta L^{*}\right)}^{2}\;+\;{\left(\Delta a^{*}\right)}^{2}\;+\;(\Delta {b^{*})}^{2}}$$

Among these, ΔL*, Δa*, and Δb* represent the color differences between the sample and the background of the standard film (L* = 88.6, a* = −1.55, b* = 1.63).

### 2.7. Powder Yield of Camellia Oil Microcapsules

After spray drying, the powder was collected from the cyclone separator and collector and weighed (m_c_). The powder yield was calculated by comparing the collected powder mass (m_c_) to the total solid mass (m_sc_) using the formula

$$\mathrm{Powder}\;\mathrm{yield}\;(\%)=\frac{m_{c}}{m_{\mathrm{sc}}}\;\times \;100$$

### 2.8. Moisture Content of Camellia Oil Microcapsules

Moisture content was calculated from the mass loss following direct drying at 105 °C and 101.3 kPa. The formula for calculating moisture content is as follows:

$$\mathrm{Moisture}\;\mathrm{content}\;\left(\%\right)\;=\;\frac{W_{1}-W_{2}}{W_{1}}\;\times \;100$$

W_1_ is the sample weight before drying (g), and W_2_ is the sample weight after drying (g).

### 2.9. Bulk Density of Camellia Oil Microcapsules

Approximately 2 g of microcapsule sample was accurately weighed and slowly transferred into a 10 mL graduated cylinder. The cylinder was then placed horizontally on the bench and gently tapped to achieve consistent powder packing. The tapped volume was recorded and divided by the sample mass to obtain the bulk density. Bulk density is calculated using the formula

$$\mathrm{Bulk}\;\mathrm{density}\;\left(\frac{g}{{\mathrm{cm}}^{3}}\right)=\frac{\mathrm{Sample}\;\mathrm{weight}\;(g)}{\mathrm{Tapped}\;\mathrm{volume}\;({\mathrm{cm}}^{3})}$$

### 2.10. Solubility of Camellia Oil Microcapsules

The solubility of camellia oil microcapsules was determined using a gravimetric method adapted from Aksoylu Özbek et al. [14]. Briefly, 0.2 g of microcapsules was dispersed in 20 mL of distilled water and stirred magnetically for 5 min. After centrifugation at 3000 rpm for 5 min, the supernatant was transferred to a pre-weighed, pre-dried Petri dish and oven-dried at 105 °C to constant weight. The solubility percentage was then calculated gravimetrically.

### 2.11. Microscopic and Particle Size Analysis of Camellia Oil Microcapsules

The specimen stage was coated with conductive carbon tape, followed by the adhesion of a small amount of camellia oil microcapsules. Excess powder was gently blown away, and the samples were sputter-coated with a gold layer. The gold-coated specimen was then positioned in the chamber of a scanning electron microscope (Hitachi S4800, Tokyo, Japan). The microstructure of the microcapsules was examined to assess surface features, including wrinkling, indentations, and particle rupture.

Particle size distribution was determined from the SEM images using Nano Measurer software (version 1.2), with three replicate analyses performed for each sample. For each analysis, three separate SEM images of the same sample were imported, 50 particles were manually measured, and the average value was calculated.

### 2.12. Oil Content Analysis of Camellia Oil Microcapsules

The total oil content and surface oil content of the microencapsulated camellia oil were evaluated. To determine the surface oil content, 4 g of each sample was accurately weighed into a 250 mL conical flask. Subsequently, 15 mL of petroleum ether was added, and the mixture was gently agitated for 10 s. The solution was then filtered, and the filtrate was collected in a 50 mL beaker that had been pre-dried to a constant weight in a 105 °C oven. The residue was transferred back to the original conical flask, washed twice with 10 mL of petroleum ether each time, and filtered after gentle shaking. The filtrates were combined in the same beaker. The solvent was evaporated in a 60 °C water bath under a fume hood, and the beaker was dried to a constant weight in a 105 °C oven for at least 1 h. After cooling, the sample was weighed. For the total oil content, 0.5 g of the powder was dissolved in 20 mL of distilled water and vortexed until fully dissolved. Then, 20 mL of hexane was added to the solution, followed by vortexing for 5 min. The mixture was centrifuged at 3500 rpm for 20 min, and the resulting supernatant was collected for the quantification of encapsulated camellia oil. The oil encapsulation efficiency (Oil-EE%) was calculated using the formula

$$\text{Oil-EE}\%=\frac{\left(\mathrm{Total}\;\mathrm{amount}\;\mathrm{of}\;\mathrm{loaded}\;\text{oil-surface}\;\mathrm{content}\;\mathrm{of}\;\mathrm{oil}\right)}{\mathrm{Total}\;\mathrm{amount}\;\mathrm{of}\;\mathrm{loaded}\;\mathrm{oil}}\;\times \;100$$

### 2.13. Fourier Transform Infrared Spectra of Camellia Oil Microcapsules

The prepared camellia oil microcapsules were placed in a Fourier transform infrared spectrometer (Spectrum 400, Perkin Elmer, Los Angeles, CA, USA). Experimental parameters were set as follows: the scanning wavelength range was 400–4000 cm^−1^, the resolution was set to 4 cm^−1^, and the cumulative number of scans was 32.

### 2.14. Thermal Stability Measurement of Camellia Oil Microcapsules

The thermal stability of the camellia oil microcapsules under nitrogen was determined by thermogravimetric analysis (SDT Q600, TA, New Castle, DE, USA). 5 mg of sample was accurately weighed into an alumina crucible. Mass loss was determined by heating the sample from 0 to 600 °C at a controlled rate of 10 °C/min, and the corresponding mass loss was recorded as a function of temperature to generate the mass loss versus temperature profile. The sample’s thermal weight loss curve was plotted by measuring its weight change.

### 2.15. Encapsulation Efficiency of Camellia Oil Microcapsules

The encapsulation efficiency of β-carotene was determined according to the method of Yin et al. with minor modifications [15]. Briefly, 0.1 g of microcapsules was mixed with 10 mL of n-hexane, vortexed for 30 s, and centrifuged at 5000 rpm for 5 min. The supernatant was collected and appropriately diluted, and its absorbance was measured at 450 nm using a UV–visible spectrophotometer. The concentration of unencapsulated β-carotene was determined from a pre-established standard calibration curve. The encapsulation efficiency was then calculated as follows:

$$\beta \text{-carotene-EE}\%\;=\;\frac{A_{0}-A_{w}}{A_{0}}\times 100$$

In the formula, A_0_ represents the initial content of β-carotene added to the oil phase; A_w_ represents the content of unencapsulated β-carotene.

### 2.16. Antioxidant Activity Assay of Camellia Oil Microcapsules

The antioxidant activity of camellia oil microcapsules was determined using the DPPH radical scavenging assay. A 0.1% solution was prepared by dissolving 0.1 g of camellia oil microcapsules in 100 mL of deionized water. The solution was then subjected to centrifugation at 8000 rpm for 5 min. Subsequently, 2 mL of the supernatant was mixed with an equal volume (2 mL) of a 0.1 mM DPPH solution. After incubation in the dark for 20 min, the absorbance of the solution was measured at λmax 517 nm using a UV–visible spectrophotometer (Lambda 465, PerkinElmer, Waltham, MA, USA). The antioxidant activity was calculated using the following formula:

$$\mathrm{DPPH}\;\mathrm{radical}\;\mathrm{scavenging}\;\mathrm{activity}(\%)\;=\;1-\frac{A_{0}-A_{1}}{A_{2}}\;\times \;100$$

A_0_ represents the absorbance of the test group, A_1_ represents the absorbance of the control group, and A_2_ represents the absorbance of the blank group.

### 2.17. In Vitro Digestion Analysis of Camellia Oil Microcapsules

During the gastric phase, NaCl was dissolved in deionized water (2 mg/mL) and adjusted to pH 2.0, and pepsin (3.2 mg/mL) was dispersed in SGF. A microcapsule solution containing 10% camellia oil was combined in equal volume with SGF. The mixture was then incubated at 37 °C under constant agitation for a duration of 2 h to simulate gastric digestion. For the small intestinal phase, the gastric phase sample was rapidly adjusted to pH 7.0 before mixing with simulated intestinal fluid containing 8.3235 mg/mL CaCl_2_, 4.8 mg/mL lipase solution, and 5 mg/mL bile salt solution. The mixture was titrated with 1 M NaOH solution and incubated for 2 h while maintaining pH 7.0, and the consumed NaOH volume was recorded and the released free fatty acids (FFAs) calculated from the titration curve using the following equation:

$$\mathrm{FFAs}\;\left(\%\right)\;=\;\frac{V_{N}\;\times \;M_{N\;}\times \;M_{L}}{2W_{L}}\;\times \;100$$

where V_N_ and M_N_ represent the consumed volume (L) and concentration (mol/L) of NaOH, respectively, while M_L_ and W_L_ denote the molecular weight (g/mol) and weight (g) of camellia oil, respectively.

Following gastric digestion and intestinal digestion, the microstructure of droplets in the solution was immediately observed using a laser confocal scanning microscope (SP8, Leica, Wetzlar, Germany).

### 2.18. Statistical Analysis

All experiments were conducted in triplicate, with experimental data expressed as mean ± standard deviation. Statistical analyses were conducted utilizing SPSS version 25 software (IBM Software, Chicago, IL, USA). Differences among groups were determined by one-way analysis of variance (ANOVA) followed by appropriate post hoc tests for multiple comparisons. The significance level was set at *p* < 0.05.

## 3. Results and Discussion

### 3.1. Rheological Properties of Camellia Oil Emulsion

**Figure 2:**
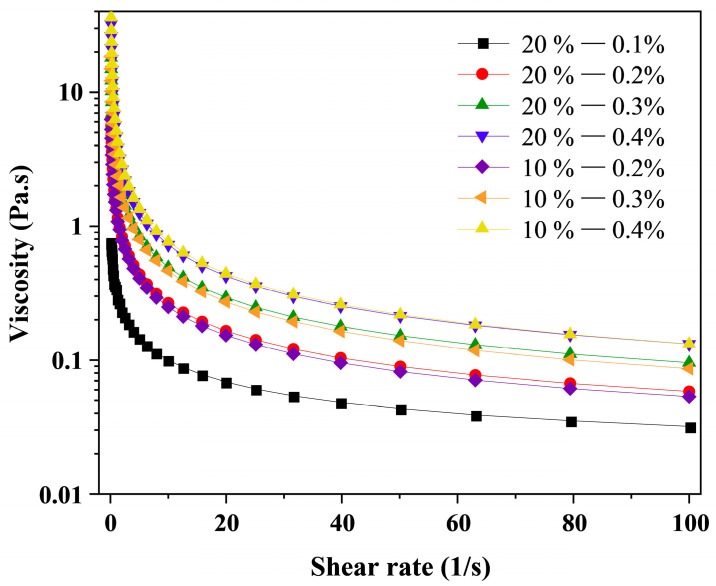
Steady-state shear curve of camellia oil emulsion. Legends indicate oil phase content (20% or 10%) and XG concentration (0.1%, 0.2%, 0.3%, or 0.4%).

The viscosity of the emulsion is a key factor influencing the properties of microencapsulated powders. The steady-state shear curve of the camellia oil emulsion shown in Figure 2 indicates that all samples exhibit pseudoplastic behavior, with viscosity decreasing sharply as shear rate increases—particularly pronounced in the low-shear-rate region—due to the disruption of the emulsion’s weak network structure [16]. As XG concentration increases, the viscosity of the camellia oil emulsion gradually rises. This is attributed to XG’s excellent thickening properties, where the formed network structure acts as a physical barrier. However, excessively high emulsion viscosity reduces the droplet formation rate during atomization and prolongs the emulsion’s exposure to high temperatures. This increases the risk of forming wet, sticky particles, leading to particle adhesion on dryer walls and reduced yield. These conditions not only impede the efficiency of the spray drying process but may also result in the generation of large particles characterized by elevated moisture content [17]. The emulsion with a 20% oil phase exhibited slightly higher viscosity than that containing 10% camellia oil. However, in preliminary spray-drying tests, the emulsion with 20% oil content showed excessively high oil content on the microcapsule surface at the same XG concentration, potentially exceeding the encapsulation capacity of the wall material. Therefore, a 10% oil phase addition rate was selected for subsequent experiments.

Table 1 presents the shear scan fitting parameters, showing that the correlation coefficient R^2^ exceeds 0.98 for all samples, confirming the applicability of the power law model to this experimental system [18]. The n values for all samples are less than 1, further validating the emulsion’s pseudoplastic fluid characteristics. Furthermore, as XG concentration increases, the n value decreases while the k value increases, indicating enhanced pseudoplasticity and elevated viscosity in the emulsion. Compared to other XG concentration groups, the emulsion stabilized with 0.1% XG exhibits the lowest viscosity and poorer stability, prone to flocculation or coalescence. As shown in Figure S1, this system demonstrates significant stratification.

### 3.2. Confocal Laser Microscopy of Camellia Oil Emulsion

**Figure 3:**
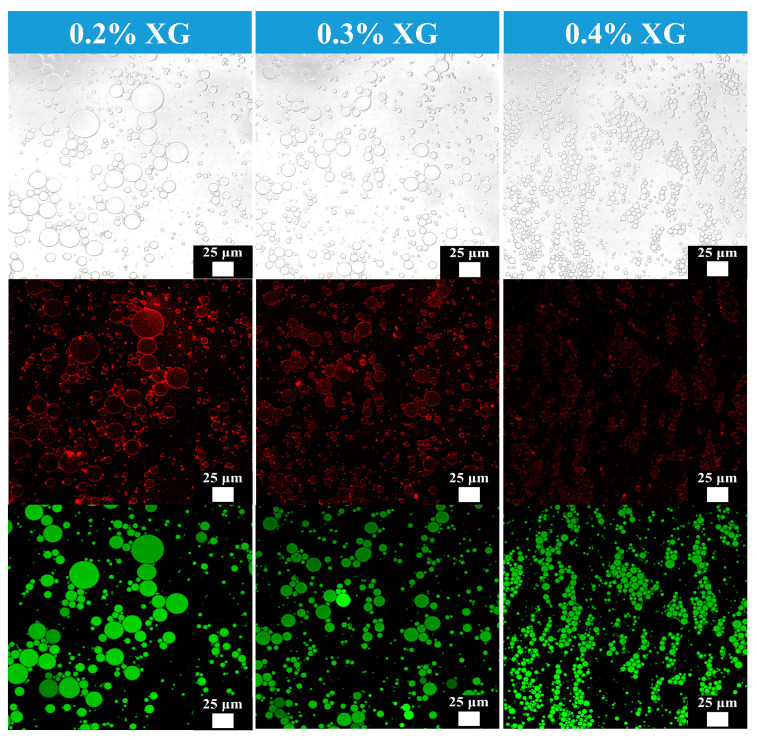
Confocal laser scanning microscope images of camellia oil emulsions prepared at different XG concentrations.

As shown in Figure 3, the morphological features of camellia oil emulsions at different XG concentrations were observed using CLSM. After staining with a mixed dye solution, XG and CAS in the aqueous phase exhibited red fluorescence, while the oil phase displayed green fluorescence. The green fluorescence of oil droplets was masked by the red fluorescent protective layer formed by XG and CAS, thereby indicating the successful formation of oil-in-water emulsion [19].

Results indicate that XG concentration significantly regulates the microstructure of camellia oil emulsions: at 0.2% XG concentration, emulsion droplets exhibit larger sizes and uneven distribution, whereas at 0.3% XG concentration, droplet numbers increase, sizes decrease, and distribution becomes more uniform. This suggests that the network structure formed by XG effectively inhibits droplet coalescence and flocculation [20]. When the XG concentration increased to 0.4%, droplets coalesced into distinct aggregates, and dispersion uniformity decreased. This may be attributed to excessive XG concentration at this level, where high-concentration XG molecular chains became entangled, triggering droplet bridging and flocculation that disrupted dispersion uniformity.

Furthermore, the observed rheological behavior and homogeneous microstructure revealed by CLSM suggest that the emulsions, particularly those stabilized with 0.3% XG, possessed adequate short-term stability. This is crucial for preventing phase separation or droplet coalescence during the pumping and atomization stages of the spray-drying process, thereby ensuring the consistent formation of microcapsules.

### 3.3. Appearance and Color Parameters of Camellia Oil Microcapsules

After spray drying at different temperatures, the camellia oil emulsion transformed into a uniformly colored milky white powder, indicating successful spray drying with no significant oil leakage. All microcapsules exhibited good dispersibility, with solubility ranging from 72.39% to 89.33%. They dissolved completely with slight stirring in distilled water at room temperature, demonstrating excellent reconstitution properties (see Figures S2 and S3). Table 2 shows the mean color attributes of the microcapsules. No significant differences were observed among the studied color attributes under different temperature treatments. As the processing temperature increased, the L* value showed a decreasing trend while the b* value exhibited an increasing trend, likely due to the Maillard reaction induced by high temperatures [21]. Excessive browning at 185 °C (high ΔE value, low L* value) positively correlated with the lower β-carotene encapsulation efficiency. This is because β-carotene itself is heat-sensitive and prone to degradation, while Maillard reaction products formed under harsh conditions may reflect a more severe environment that compromises the integrity of the capsule wall or core material. Negative a* and positive b* values indicated that the microcapsules exhibited pale green and yellow hues. Furthermore, as XG concentration increased, the L* value of the camellia oil powder rose while the a* and b* values decreased. This suggests that adding XG helps regulate the color quality of camellia oil microcapsules, playing a positive role in optimizing the product’s appearance characteristics.

### 3.4. Powder Yield, Moisture Content, and Bulk Density of Camellia Oil Microcapsules

Preliminary experiments indicate that when the spray drying inlet temperature is below 145 °C, the camellia oil emulsion cannot achieve an acceptable yield. Therefore, the inlet temperature for subsequent spray drying was set between 145 °C and 185 °C. As shown in Table 3, the yield of camellia oil emulsion first increases and then decreases with rising inlet temperature, reaching a maximum of 53.68% at 165 °C. This variation may be attributed to the differing surface oil content of the microcapsules, as leaked oil can promote particle adhesion to the spray dryer wall [22]. Notably, the addition of excessive XG (0.4%) caused powder particles to adhere more readily to the wall, resulting in reduced yield and increased waste.

Moisture content constitutes a critical quality attribute influencing the flowability and storage stability of microencapsulated powders, with values below 4% generally regarded as indicative of satisfactory storage stability. In Table 3, the moisture content of all microcapsules was below 2.84%, attributed to colloidal particles enhancing water-binding capacity and maintaining moisture levels [23]. Moreover, bulk density constitutes a vital attribute of spray-dried microcapsules due to its significant impact on their economic value and functional performance throughout storage, processing, packaging, and distribution stages. Research indicates that bulk density increases with rising inlet temperature but decreases with higher XG concentration. These findings likely stem from XG’s ability to reduce surface oil content, inhibit powder agglomeration, enhance powder flowability, and promote powder loosening—resulting in higher loose bulk density [8].

### 3.5. SEM and Particle Size Distributions of Camellia Oil Microcapsules

**Figure 4:**
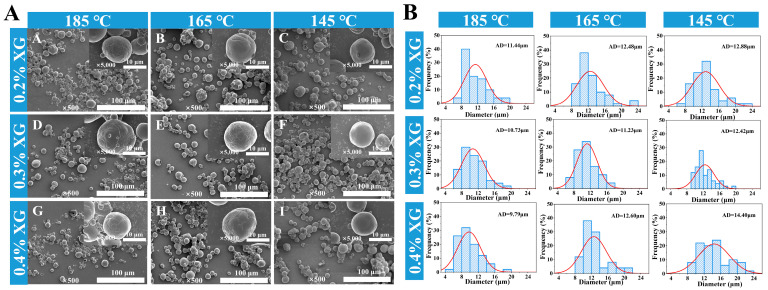
Scanning electron microscope images (**A**) and particle size frequency distribution of camellia oil microcapsules (**B**).

Drying temperature significantly affects the morphological characteristics of camellia oil microcapsules. As shown in Figure 4A, all microcapsule particles exhibit spherical shapes: under 145 °C drying conditions, the microcapsule surfaces remain smooth with intact structures; however, when the temperature rises to 185 °C, the microcapsule surfaces become uneven and even exhibit noticeable cracking. This phenomenon stems from excessive temperatures accelerating rapid dehydration and contraction at the interface where protein and polysaccharide components assemble. This process diminishes the surface elasticity of microcapsules, resulting in the development of cracks and structural damage [24]. Additionally, as XG concentration increases, some microcapsules exhibit aggregation. This result aligns with the flocculation observed in emulsion droplets prior to drying.

The particle size distribution, as determined from microscopic images using image analysis software, is shown in Figure 4B. At a comparatively low drying temperature of 145 °C, the microcapsule particles exhibited an average size of approximately 12 μm. When the drying temperature was increased to 185 °C, most microcapsules had particle sizes below 10 μm, with a narrower size distribution. This occurs because at specific drying temperatures, the rate of water transfer from the microcapsule interior to the surface reaches equilibrium with the rate of surface moisture evaporation. Excessively high temperatures, however, cause rapid and massive water evaporation, leading to instantaneous shrinkage of the droplets and ultimately forming particles with relatively smaller diameters [25].

### 3.6. Oil Content of Camellia Oil Microcapsules

**Figure 5:**
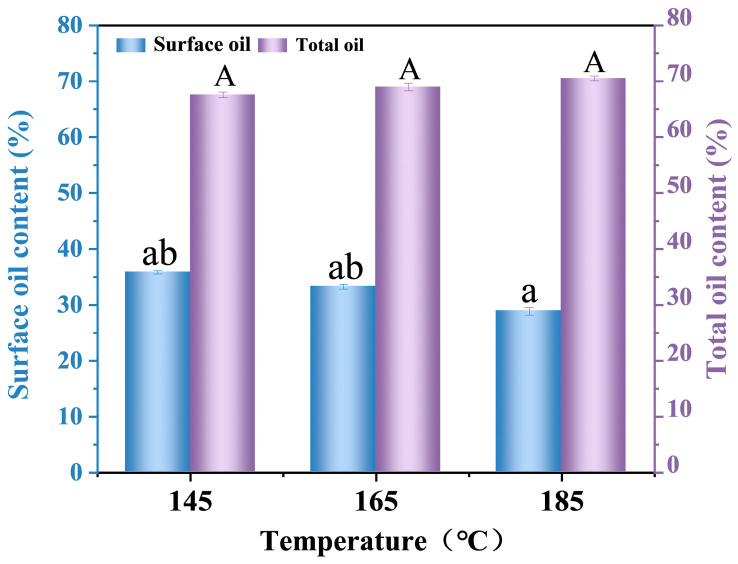
Surface oil content and total oil content of 0.3% XG-concentrated camellia oil microcapsules at different drying temperatures. Bars labeled with the same uppercase letters indicate no significant difference in total oil content among samples at different temperatures (*p* > 0.05). Bars labeled with the same lowercase letters indicate no significant difference in surface oil content among samples at different temperatures (*p* > 0.05). Data were analyzed using Welch’s ANOVA followed by Games–Howell post hoc tests.

As shown in Figure 5, spray drying technology was employed to examine the impact of different temperatures on the oil encapsulation efficiency of camellia oil microcapsules prepared with 0.3% XG. Results showed oil encapsulation efficiency ranging from 46.94% to 59.05%, comparable to the camellia oil emulsions prepared by Li et al. using vacuum drying (encapsulation efficiency of 49.68–66.42%) [26], demonstrating that the microcapsules prepared in this experiment possess certain oil-holding capacity. Regarding the temperature influence mechanism: At 145 °C, the drying process was relatively mild. The aqueous phase carrier structure synergistically formed by CAS and XG provided stable encapsulation for the oil phase. However, due to incomplete drying, some oil tended to adhere to the particle surface, resulting in elevated surface oil content. As drying temperature increases, accelerated moisture evaporation effectively reduces surface oil migration and evaporation, resulting in decreasing surface oil content. At 185 °C, microcapsules rapidly dehydrate and exhibit localized damage. While high temperatures promote carrier densification, heightened molecular thermal motion increases rupture risk. This demonstrates that precise temperature control is critical for ensuring microcapsule product quality.

### 3.7. FTIR Analysis of Camellia Oil Microcapsules

**Figure 6:**
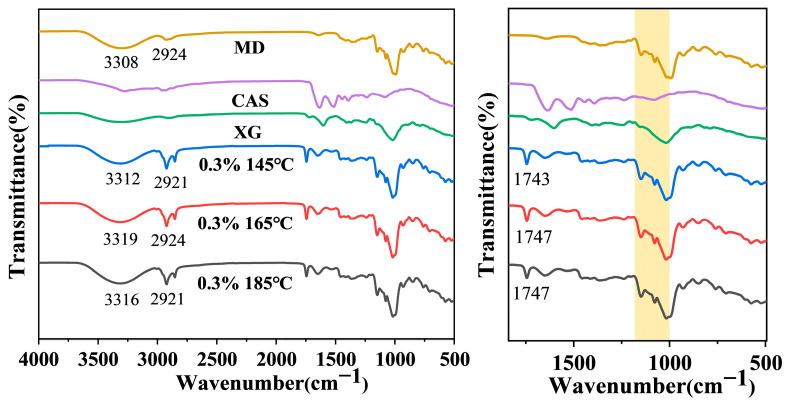
Full Fourier transform infrared spectra (4000–400 cm^−1^) and magnified spectra (1800–500 cm^−1^) of 0.3% XG concentration camellia oil microcapsules and their components at different drying temperatures. The yellow strip indicates the C-O stretching vibration peak in the 1000–1200 cm^−1^ region.

FTIR spectroscopy was employed to investigate the intermolecular interactions and structural changes in the microcapsule powder. As illustrated in Figure 6, all microcapsule samples exhibited a new absorption peak around 1740 cm^−1^, attributable to the C=O stretching vibration of fatty acid ester groups in camellia oil. This result confirms the presence of camellia oil in the microcapsule powder, likely due to minor oil leakage during spray drying, resulting in oil residues on the microcapsule surface. The low surface oil content and high encapsulation efficiency of β-carotene further confirm that the majority of the oil has been incorporated into the wall matrix rather than exposed on the surface [17]. Following the incorporation of MD as a wall material, the microcapsules exhibited broadened -OH stretching vibration peaks near 3300 cm^−1^. These peaks became more pronounced with increasing temperature, indicating that elevated temperatures intensified intermolecular interactions. A distinctive C-O stretching vibration peak also appeared in the 1000–1200 cm^−1^ region, consistent with MD’s signature peak, indicating that the added MD coated the droplet surface stabilized by XG and CAS. Furthermore, an absorption peak observed around 2900 cm^−1^ is attributed to asymmetric C-H stretching vibrations, consistent with conclusions from relevant literature [27]. It is noteworthy that the characteristic peak of XG and CAS at 1600 cm^−1^ observed in the microcapsules is attributable to the wall material forming the continuous matrix of the microcapsules, and its clear detection is an expected phenomenon.

### 3.8. Thermal Stability of Camellia Oil Microcapsules

**Figure 7:**
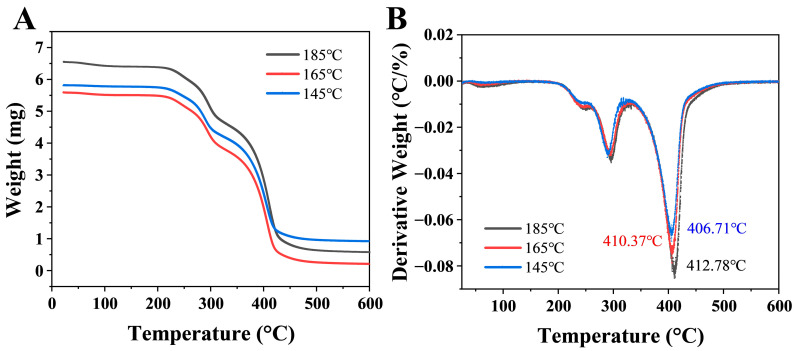
TGA (**A**) and DTG (**B**) curves of camellia oil microcapsules at 0.3% XG concentration at different drying temperatures.

TGA and DTG were employed to evaluate the thermal stability of microcapsules. As shown in Figure 7A, the thermal weight loss process of microcapsule samples primarily exhibits three distinct stages with increasing temperature: Stage I occurs below 220 °C, characterized by relatively slow mass change primarily attributed to water evaporation, release of volatile substances, and partial desorption of small molecules. This suggests the existence of both free water and trace quantities of bound water within the wall material. The second stage (220–400 °C) exhibits a steep downward trend in the TGA curve, primarily due to the decomposition of the majority of the wall material, leading to microcapsule shell rupture. The third stage (400–600 °C) is characterized by the continued decomposition of the wall material. This phase is primarily associated with the rapid volatilization of camellia oil and the complete cessation of thermal decomposition reactions [28]. Pure camellia oil begins to significantly volatilize and decompose at temperatures around 300–400 °C. The higher decomposition temperatures exhibited by these microcapsules indicate that the wall material effectively protects the encapsulated oil, thereby enhancing its thermal stability. As shown in the DTG curve of Figure 7B, the maximum thermal degradation temperatures of microcapsules prepared at different spray-drying temperatures were 412.78, 410.37, and 406.71 °C, respectively, exhibiting an overall upward trend with increasing temperature. Generally, a higher thermal degradation temperature often indicates a more compact molecular structure.

### 3.9. Encapsulation Efficiency and Antioxidant Activity of Camellia Oil Microcapsules

**Figure 8:**
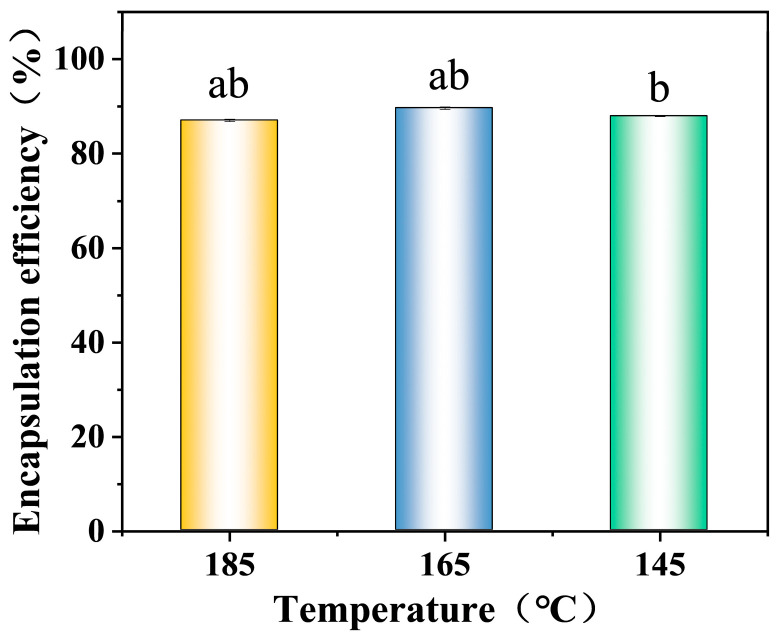
β-carotene encapsulation efficiency of 0.3% XG concentration camellia oil microcapsules at different drying temperatures. Columns labeled with the same lowercase letter indicate no significant difference (*p* > 0.05). Data were analyzed using Welch’s ANOVA followed by Games–Howell post hoc tests.

This study systematically evaluated the encapsulation performance of microcapsules prepared from 0.3% XG-stabilized emulsions at different drying temperatures, using β-carotene as the model encapsulate. Figure 8 shows that the encapsulation efficiencies of β-carotene in microcapsules prepared at different drying temperatures all exceeded 85%, indicating that β-carotene was well encapsulated within the microcapsules after spray drying [29]. Specifically, microcapsules prepared at 165 °C exhibited the highest encapsulation efficiency at 89.6%, while those prepared at 145 °C and 185 °C showed reduced encapsulation efficiencies of 87.9% and 87.0%, respectively. This discrepancy stems from two factors: lower temperatures slow the evaporation rate of water in the emulsion, causing partially undried emulsion to adhere to the inner walls of the drying equipment, thereby reducing microencapsulated powder yield and consequently lowering the encapsulation efficiency. Conversely, higher temperatures induce chemical degradation of β-carotene and also cause some β-carotene to adsorb onto the drying chamber walls, further decreasing the encapsulation efficiency [25]. Thus, drying temperature significantly regulates the encapsulation efficiency of β-carotene.

Additionally, as shown in Supplementary Figure S4, microcapsules prepared at 165 °C exhibited the highest radical scavenging rate of approximately 74.80 ± 0.34%, demonstrating strong antioxidant activity. This is attributed to the increased encapsulation of antioxidant-active β-carotene within the microcapsules at this temperature, enabling greater release of active components and enhanced scavenging efficacy.

### 3.10. In Vitro Digestion of Camellia Oil Microcapsules

**Figure 9:**
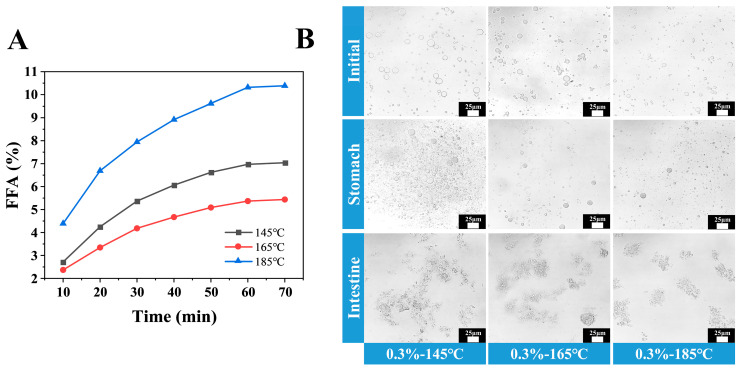
Release curves of free fatty acids during the small intestinal phase (**A**) and microscopic characteristics (**B**) from 0.3% XG concentration camellia oil microcapsules at different drying temperatures.

The digestive properties of encapsulated camellia oil were evaluated utilizing an in vitro digestion model that simulates the conditions of the stomach and small intestine. The microscopic appearance of camellia oil microcapsules at different drying temperatures during digestion is shown in Figure 9A: Compared to the initial microcapsules, partial hydrolysis occurred during the gastric phase. During the intestinal phase, microcapsules were extensively digested, forming flocculent oil droplets with only a few residual droplets remaining. As drying temperature increased, the digestion rates of camellia oil microcapsules accelerated synchronously in both the gastric and intestinal phases [30]. Furthermore, the free fatty acid (FFA) release curve in Figure 9B further confirmed that all samples exhibited the fastest hydrolysis rates within the first 50 min, gradually slowing down during subsequent hydrolysis periods. The rapid FFA generation stems from sufficient interstitial space between colloidal particles adsorbed at the oil-water interface. This allows bile salts and lipase to diffuse to the lipid droplet surface, initiating hydrolysis [31]. Notably, higher drying temperatures correlated with increased FFA release. This finding can be explained by the “enzyme contact mechanism”: elevated spray-drying temperatures reduce microcapsule oil content (as indicated by earlier oil content data), thereby increasing the surface area of fat enzymes exposed to the oil phase. This facilitates thorough hydrolysis in the small intestine, releasing substantial amounts of FFA [8].

## 4. Conclusions

This study successfully prepared camellia oil microcapsules using XG and CAS as emulsion templates combined with spray drying, identifying the most favorable preparation conditions and validating the product’s application potential. Results indicate that 0.3% XG ensures uniform emulsion droplet dispersion while balancing microcapsule bulk density and solubility (solubility range: 72.39–89.33%). It also enhances product appearance by increasing lightness (L*) and reducing redness (a*) and yellowness (b*). Spray drying temperature directly impacts microcapsule quality, with 165 °C being optimal. At this temperature, microcapsule yield reached 53.68%, moisture content was <2.84%, and both β-carotene encapsulation efficiency (89.6%) and DPPH radical scavenging rate (74.80 ± 0.34%) were maximized. FTIR confirmed successful encapsulation of camellia oil, while TGA demonstrated excellent thermal stability (maximum thermal degradation temperature exceeding 400 °C). In vitro digestion experiments indicated efficient release of free fatty acids during the small intestinal phase. In summary, this study established 0.3% XG concentration and 165 °C drying temperature as optimal conditions for camellia oil microencapsulation. The resulting product exhibits excellent stability, functional activity, and processing adaptability, effectively addressing core challenges in camellia oil storage and application. This study establishes a theoretical framework for the high-value utilization of camellia oil and offers a reference model for microencapsulation research on similar oils. Subsequent experiments can prepare microcapsules with a more concentrated particle size distribution by optimizing homogenization parameters and atomizer performance.

## Figures and Tables

**Table 1:** Power law model parameters for emulsions stabilized by different concentrations of XG.

Samples	Oil Phase (%)	k (Pa·s^n^)	n	R^2^
0.1%XG	20	0.314	0.577	0.986
0.2%XG	20	1.395	0.329	0.999
0.3%XG	20	2.860	0.205	0.999
0.4%XG	20	4.593	0.158	0.999
0.2%XG	10	1.288	0.314	0.999
0.3%XG	10	2.770	0.184	0.999
0.4%XG	10	5.083	0.154	0.999

**Table 2:** Color coordinates (L*, a*, b* and ΔE) of microencapsulated camellia oil powder at different drying temperatures.

Samples	Inlet Air Temperature (°C)	L	a*	b*	ΔE
0.2%XG	145 °C	93.15 ± 0.10 ^cA^	−0.61 ± 0.02 ^bA^	5.14 ± 0.05 ^aB^	5.82 ± 0.09 ^bA^
	165 °C	89.88 ± 0.15 ^aA^	−0.85 ± 0.02 ^aA^	6.74 ± 0.12 ^bC^	5.31 ± 0.15 ^aA^
	185 °C	91.24 ± 0.06 ^bA^	−0.83 ± 0.01 ^aA^	9.42 ± 0.15 ^cC^	8.26 ± 0.13 ^cB^
0.3%XG	145 °C	94.32 ± 0.14 ^cB^	−0.62 ± 0.02 ^cA^	5.73 ± 0.05 ^aC^	7.31 ± 0.25 ^bB^
	165 °C	93.62 ± 0.28 ^bB^	−0.72 ± 0.02 ^bB^	5.08 ± 0.13 ^aB^	6.58 ± 0.21 ^aB^
	185 °C	93.23 ± 0.02 ^aB^	−0.77 ± 0.01 ^aB^	7.50 ± 0.30 ^bB^	7.52 ± 0.21 ^bA^
0.4%XG	145 °C	95.75 ± 0.05 ^bC^	−0.55 ± 0.03 ^bB^	4.48 ± 0.04 ^aA^	7.76 ± 0.05 ^bC^
	165 °C	95.66 ± 0.05 ^bC^	−0.56 ± 0.01 ^bC^	4.40 ± 0.07 ^aA^	7.65 ± 0.06 ^bC^
	185 °C	94.83 ± 0.11 ^aC^	−0.63 ± 0.01 ^aC^	5.57 ± 0.12 ^bA^	7.42 ± 0.11 ^aA^

Note: Different upper/lower case letters in the graph indicate significant differences between samples with different XG concentrations at the same temperature or different temperatures at the same XG concentration (*p* < 0.05).

**Table 3:** Effect of XG concentration and spray drying temperature on yield, moisture content and bulk density of oil powder microcapsules.

Samples	Inlet Air Temperature (°C)	Powder Yield (%)	Moisture Content (%)	Bulk Density (g/cm^3^)
0.2%XG	185 °C	49.16 ± 0.40 ^cB^	2.68 ± 0.03 ^cC^	0.32 ± 0.007 ^aB^
	165 °C	46.52 ± 0.28 ^bB^	2.39 ± 0.02 ^bB^	0.46 ± 0.018 ^bC^
	145 °C	43.59 ± 0.39 ^aB^	1.77 ± 0.02 ^aA^	0.46 ± 0.010 ^bC^
0.3%XG	185 °C	49.51 ± 0.43 ^bB^	2.29 ± 0.04 ^bB^	0.31 ± 0.004 ^aA^
	165 °C	53.68 ± 0.38 ^cC^	1.89 ± 0.03 ^aA^	0.36 ± 0.003 ^bB^
	145 °C	34.96 ± 0.82 ^aB^	1.92 ± 0.04 ^aB^	0.40 ± 0.007 ^cB^
0.4%XG	185 °C	28.07 ± 0.43 ^aA^	2.08 ± 0.07 ^aA^	0.31 ± 0.002 ^aA^
	165 °C	36.97 ± 0.25 ^bA^	2.49 ± 0.04 ^bC^	0.33 ± 0.007 ^bA^
	145 °C	27.60 ± 0.51 ^aA^	2.84 ± 0.04 ^cC^	0.35 ± 0.009 ^cA^

Note: Different upper/lower case letters in the graph indicate significant differences between samples with different XG concentrations at the same temperature or different temperatures at the same XG concentration (*p* < 0.05).

## Data Availability

The original contributions presented in the study are included in the article/Supplementary Materials, and further inquiries can be directed to the corresponding authors.

## Supplementary Materials

The following supporting information can be downloaded at https://www.mdpi.com/article/10.3390/foods14213610/s1, Figure S1: Appearance of camellia oil emulsions prepared with different oil phases and varying XG concentrations. Figure S2: Appearance of camellia oil microcapsules prepared at different drying temperatures and varying XG concentrations. Figure S3: Solubility of camellia oil microcapsules prepared at different drying temperatures and varying XG concentrations. Figure S4: Antioxidant capacity of camellia oil microcapsules prepared at different drying temperatures and varying XG concentrations.
