# Supplementary material for: Preparation and Characterization of Camellia Oil Microcapsules Using Spray Drying Coupled with Sodium Caseinate/Xanthan Gum-Stabilized Emulsion Template
Source: Foods. 2025 Oct 23;14(21):3610. doi: 10.3390/foods14213610 (PMC12610554; doi:10.3390/foods14213610)
Supplement: Supplementary file 1 [file foods-14-03610-s001.zip › foods-3901574-supplementary.pdf]

Preparation and characterization of camellia oil microcapsules using spray drying coupled with sodium caseinate/xanthan gum stabilized emulsions template

Wei Xu[a*], Lala Li[a], Yingying Xin[a], Jiawei Xue[a], Lihua Zhang[a], Bakht Ramin Shah[b]

[a] College of Life Science, Xinyang Normal University, Xinyang, 464000, China

[b] DRIFT-FOOD Centre, Faculty of Agrobiology, Food and Natural Resources, Czech University of Life Sciences, Prague 16500, Czech Republic

*Corresponding author:

Wei Xu, xuwei@xynu.edu.cn(W Xu)

This file includes:

Fig. S1 Appearance of camellia oil emulsions prepared with different oil phases and varying XG concentrations.

Fig. S2 Appearance of camellia oil microcapsules prepared at different drying temperatures and varying XG concentrations.

Fig. S3 Solubility of camellia oil microcapsules prepared at different drying temperatures and varying XG concentrations.

Fig. S4 Antioxidant capacity of camellia oil microcapsules prepared at different drying temperatures and varying XG concentrations.

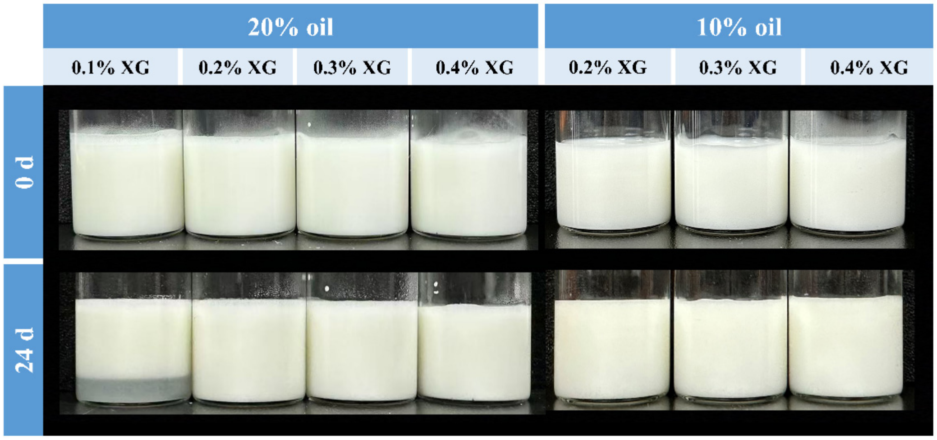

Fig. S1 Appearance of camellia oil emulsions prepared with different oil phases and varying XG concentrations.

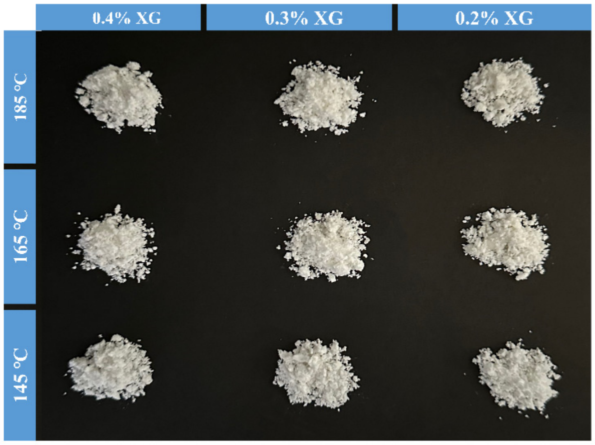

Fig. S2 Appearance of camellia oil microcapsules prepared at different drying temperatures and varying XG concentrations.

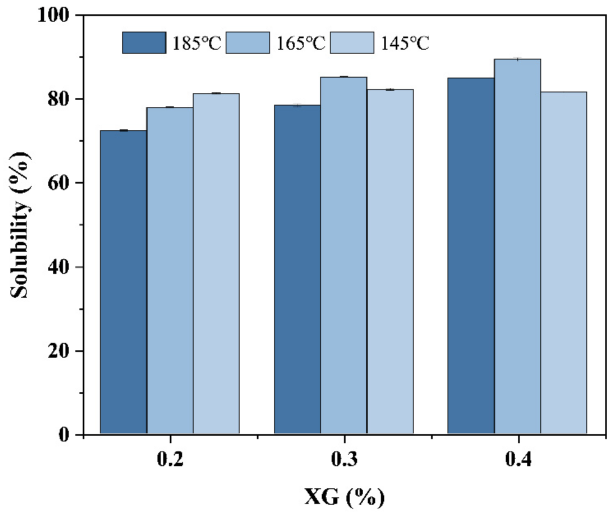

Fig. S3 Solubility of camellia oil microcapsules prepared at different drying temperatures and varying XG concentrations.

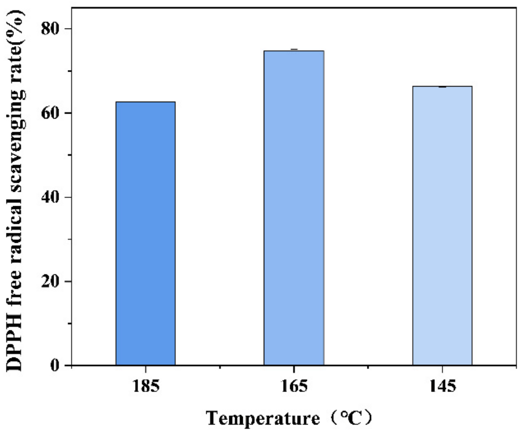

Fig. S4 Antioxidant capacity of camellia oil microcapsules prepared at different drying temperatures and varying XG concentrations.
